# Comparing the Prevalence of Psychiatric Disorders in Cohorts of Children Born Extremely Preterm in 1995 and 2006: The EPICure Studies

**DOI:** 10.1016/j.jaacop.2024.02.005

**Published:** 2024-09

**Authors:** Jennifer Larsen, Josephine Holland, Puja Kochhar, Dieter Wolke, Elizabeth S. Draper, Neil Marlow, Samantha Johnson

**Affiliations:** a University of Leicester, Leicester, United Kingdom; b University of Nottingham Nottingham, United Kingdom; c University of Warwick, Coventry, United Kingdom; d University College London, London, United Kingdom

**Keywords:** infant, extremely premature, mental disorders, psychiatric diagnosis, Developmental and Wellbeing Assessment (DAWBA)

## Abstract

**Objective:**

This study aimed to identify the prevalence of psychiatric disorders in 2 population-based cohorts of children born extremely preterm (EP) 11 years apart to ascertain whether psychiatric outcomes have changed over time following improved survival of EP children.

**Method:**

In the EPICure2 study, 200 children born EP (22-26 weeks’ gestation) in England in 2006 were assessed at 11 years of age alongside 143 term-born children. Children were assessed using the Developmental and Wellbeing Assessment (DAWBA). *DSM-IV* diagnoses were assigned by clinical psychiatrists for 145 EP and 98 term-born children. Outcomes were compared between a subsample of children from the EPICure2 cohort (2006, n = 76) and the earlier-born EPICure (1995, n = 161) cohort born at 22 to 25 weeks’ gestation in England.

**Results:**

EP children in EPICure2 were significantly more likely than term-born children to have any psychiatric disorder (39.3% vs 3.1%; adjusted odds ratio [OR] = 15.1, 95% CI = 4.4-51.1), emotional disorders (14.6% vs 2.0%; OR = 7.3, 95% CI = 1.6-32.7), conduct disorders (6.3% vs 0.0%, *p =* .01), attention-deficit/hyperactivity disorder (ADHD, 21.9% vs 2.6%; OR = 7.2; 95% CI = 1.5-33.6), and autism spectrum disorder (ASD, 18.9%; vs 0.0%, *p <* .001). There was no significant difference in the rates of any psychiatric disorder between EP children in the EPICure2 and EPICure cohorts.

**Conclusion:**

EP children remain at increased risk for psychiatric disorders at 11 years of age compared with term-born peers. Increased survival has not translated into improved psychiatric outcomes. Health care professionals need to be aware of this ongoing risk when caring for children born preterm.

## Plain language summary

Medical advances have improved the survival of extremely preterm children, but whether long-term outcomes have also improved is unclear. This study compared rates of psychiatric disorders at age 11 years among 76 children born extremely preterm in 1995 and 161 children born extremely preterm in 2006, to identify trends in psychiatric outcomes. Results showed that children born extremely preterm in 2006 were more likely than term children to have psychiatric disorders (39.3% vs 3.1%). There was no difference in rates of disorders between children born extremely preterm in 2006 and 1995. These results suggest that there has been no improvement in psychological outcomes for extremely preterm children, and healthcare professionals should continue to monitor for psychiatric disorders in this population.

Children born extremely preterm (EP) are at increased risk for a range of long-term adverse outcomes including neurodevelopmental disability,^1^ cognitive impairment,^2^ and behavioral difficulties.^3^^,^^4^ The risk of mental health problems and psychiatric disorders during childhood and adolescence is also higher for those born EP relative to those born at term, in particular for emotional disorders, attention-deficit/hyperactivity disorder (ADHD), and autism spectrum disorder (ASD).^5^, ^6^, ^7^, ^8^

The majority of studies investigating psychopathology following preterm birth have used screening measures and have focused on symptom burden. Results from key prospective birth cohort studies show that EP children have greater levels of symptoms than term-born children or when compared with levels expected in the general population.^9^, ^10^, ^11^, ^12^, ^13^ Fewer studies have used diagnostic evaluations to investigate the risk for psychiatric disorders following preterm birth. A meta-analysis of 5 diagnostic studies found that children and young adults born preterm were at increased risk for any psychiatric disorder (pooled odds ratio [OR] = 3.66; 95% CI = 2.57-5.21) and anxiety/depression (pooled OR = 2.86; 95% CI = 1.73-4.73); however, the studies pooled included a variety of gestational age/birthweight cohorts, and the majority were born before the 1990s.^8^ More recent meta-analyses have reported that children born very preterm/very low birth weight (VP/VLBW) had a 2.25 (95% CI = 1.56-3.26)^6^ and 3.3 (95% CI = 2.0-5.6)^14^ higher odds of ADHD diagnosis compared with term-born children, and those born EP/extremely low birth weight (ELBW) were at even greater risk (OR = 4.05, 95% CI = 2.38-6.87).^6^ Similarly, a meta-analysis by Agrawal *et al.*^7^ calculated a pooled prevalence of ASD of 7% in preterm-born children with a median gestational age of 28 weeks (range, 25-31 weeks), a rate much higher than that in the general population. However, they did not find an association between the prevalence of ASD and gestational age or birthweight. An individual participant meta-analysis of VP/VLBW individuals between 6 and 32 years of age also found increased risk for ASD, ADHD, anxiety, and mood disorders.^15^

The rates of psychiatric disorders have also been investigated in longitudinal cohorts of VP/EP children. The Victorian Infant Brain Studies (VIBeS) assessed children born at less than 30 weeks of gestation or with birthweight less than 1250 g (VP/VLBW) between 2001 and 2003 at 7 and 13 years of age using the Development and Well-Being Assessment (DAWBA). Compared to term-born children, children born VP/VLBW were more than 3 times as likely to have a psychiatric disorder at 7 years of age^16^ and almost 6 times more likely at 13 years.^17^ VP/VLBW children also had higher rates of anxiety disorders, ADHD, and ASD, but between-group differences were not statistically significant.^16^^,^^17^ Similar to the VIBeS findings, EP children in the EPICure cohort born between 22 and 25 weeks of gestation in the United Kingdom and Ireland in 1995 were 3 times more likely to have a diagnosis of a psychiatric disorder at age 11 years than term-born controls (OR = 3.2; 95% CI = 1.7-6.2).^18^ EP children were at increased risk for ADHD, anxiety disorders, and ASD. At age 15 years, 34% of EP children in the Extremely Low Gestational Age Newborns (ELGAN, <28 weeks of gestation) cohort had 1 or more psychiatric disorders; however, this study lacked a term-born comparison group.^19^

Findings in late adolescence/early adulthood are slightly different. The EPICure cohort at 19 years of age were assessed using the Clinical Interview Schedule—Revised (CIS-R), which found no significant differences in the prevalence of psychiatric diagnoses between EP and term-born young adults.^20^ However, EP young adults had higher symptom scores for anxiety/depression and attention problems. Likewise, no differences were reported between an Australian cohort of EP/ELBW young adults born in 1991/1992 and normal-birthweight controls at 18 years in psychiatric diagnoses made using the Structured Clinical Interview for *DSM-IV* Disorders, but higher rates of ADHD were reported using a screening measure.^21^ These results suggest that EP birth continues to predispose individuals to mental health difficulties throughout childhood and adolescence, although further research is needed to ascertain whether the increased risk of disorders continues in early adulthood.

Although advances in neonatal care have led to improved survival for EP children, there has been concern as to how this would affect longer-term outcomes as more immature and sicker infants survive.^22^^,^^23^ To date, improvements in survival have not been mirrored by improvements in longer-term outcomes. A recent meta-analysis of neurodevelopmental outcomes at 18 to 36 months in EP infants from same-center consecutive cohort studies with at least 2 cohorts born after 1990 found no significant improvement in outcomes over time.^24^ However, controlling for gestational age revealed a 3.8% improvement in neurodevelopmental outcomes, which they reported as mainly attributable to improvements in outcomes for infants born at 25 and 26 weeks of gestation. However, consecutive cohort studies of EP-born children in later childhood, assessed at 8 to 11 years of age, have found that neurodevelopmental, cognitive, or educational outcomes have not changed over time, or may even be worse,^25^^,^^26^ with health-related quality of life having deteriorated.^27^

To our knowledge, there are no studies that have investigated change in psychiatric diagnoses between 2 prospective cohorts of EP children born in different eras. In this paper, we present the prevalence of psychiatric disorders at 11 years of age in children born at 22 to 26 weeks of gestation in England in 2006 (EPICure2 cohort) compared with term-born children. We then compare the prevalence of psychiatric disorders between a subsample of the EPICure2 cohort of children born at 22 to 25 weeks of gestation with our earlier EPICure cohort of children born EP in England in 1995, to investigate whether there has been any change over time. We hypothesized that EP children in EPICure2 would have higher rates of psychiatric disorders than their term-born peers, and that there would be no significant reduction in psychiatric disorders among EP children in the EPICure2 cohort compared with those in the EPICure cohort.

## Method

### Population

The EPICure2 cohort comprises all births between 22 and 26 completed weeks of gestation in England in 2006. The cohort was recruited at birth^28^ and has been followed up at 3 years^29^ and 11 years of age.^25^ For the 11-year study, a geographical subsample was identified based around the research assessment teams and included 17 of the 45 neonatal units and networked hospitals that provided care at birth in 2006. This was necessary because of the size of the cohort for practical and financial reasons. Of the 1,041 survivors to discharge from neonatal care, 482 were invited to participate in the 11-year assessment.

The first EPICure cohort comprised all births between 22 and 25 completed weeks of gestation in all maternity units in Great Britain and Ireland between March 1 and December 31, 1995. For the present study, the 2 cohorts are compared for consistency using a subsample of children born between 22 and 25 completed weeks of gestation to mothers residing in England. The children born extremely preterm are referred to as “EP children” throughout.

A comparison group of term-born children (born ≥37 weeks of gestation) were recruited for both cohorts from classmates of the EP children in mainstream schools.^25^^,^^30^ These children were matched for age (±3 months) and sex where possible. Comparison children were identified by the EP child’s parent(s) if the EP child was home educated using the same matching criteria where possible. No term-born comparison children were recruited for EP children attending special schools. Further information on the EPICure cohorts and recruitment can be found in Marlow *et al.*^25^

### Measures

The Development and Well-Being Assessment (DAWBA) is a diagnostic interview that covers emotional, hyperactivity, autism spectrum, and conduct disorders (www.dawba.info).^31^ Parents completed the parent interview either online or by telephone with a researcher [J.L.] and teachers completed the teacher DAWBA via a paper questionnaire. Data were reviewed by 2 child and adolescent psychiatrists [J.H. and P.K.] and *DSM-IV* research diagnoses assigned based on information from the DAWBA, IQ score (Kaufman-Assessment Battery for Children 2^nd^ Edition,)^32^ and scores from the Social Communication Scale (SCQ)^33^ and Du Paul Attention-Deficit/Hyperactivity Disorder Rating Scale 5.^34^ The process used for assigning DAWBA diagnoses was the same as used previously for the EPICure cohort.^18^ Any discrepancies were resolved by consensus. As well as individual diagnoses, disorders were grouped into summary outcomes: any emotional disorder (anxiety disorders, mood disorders), any ADHD (combined, inattentive, and hyperactivity–impulsivity subtypes), any ASD (including autism, Asperger syndrome, and ASD not otherwise specified), any conduct disorder (including oppositional defiant disorder, conduct disorder) and any psychiatric disorder (any of the above and tic disorders, eating disorders, social disorders, psychosis, or stereotypic disorder). The disorders included within each of these summary categories and any psychiatric disorder can also be seen in Figure S1 (available online).

As part of the wider study, children participated in a clinical and neuropsychological assessment by a pediatrician and psychologist, and data on neurodevelopmental impairment were collected. Severe disability was defined as one or more of the following: Mental Processing Index >3 SD below term-born mean (<67) using the Kaufman-Assessment Battery for Children (K-ABC), Gross Motor Function Classification System (GMFCS)/Manual Ability Classification System (MACS) ≥3, no useful hearing with aids, no useful vision or sees only gross light/movement. The Index of Multiple Deprivation (IMD) is a widely used measure of socioeconomic deprivation in England. Areas across the country are ranked from most deprived (decile 1) to least deprived (decile 10) based on housing, environment, income, crime, housing health, and education.^35^ Population data from the time point closest to assessment for each cohort were used.

Demographic and perinatal characteristics were also available for children born EP in both cohorts and included gestational age, birthweight, multiple births, maternal age, IMD at birth, and maternal ethnicity.

### Data Processing and Statistical Analysis

Data were collected via the DAWBA website and downloaded into SPSS Statistics for Windows v28.0 (IBM Corp.).^36^ All analyses were conducted within the University College London Data Safe Haven or on anonymized data.

Descriptive statistics were used to summarize demographic data, response rates, and group characteristics of the EP and term-born children within the 2 cohorts. Drop-out analyses were carried out to ensure that individuals with complete DAWBA data did not differ significantly from those without.

First, outcomes were compared between EP and term-born children in the EPICure2 cohort. Binary logistic regression analyses were used to model differences in rates of psychiatric diagnosis between the groups. Models included adjustment for the following confounders: sex, IMD at 11 years of age, and severe disability. Children with missing data for confounders were excluded from the analysis (EP, n = 5; term, n = 3). Results are presented as odds ratios (OR) with 95% CIs. All *p* values reported are 2-tailed (α = 0.05). Sensitivity analyses excluding children with severe cognitive impairment (IQ <70) were conducted. Where the prevalence of disorder in the term-born group were zero and logistic regression was therefore not possible, χ^2^ and Fisher’s exact tests were used to assess between-group differences.

Second, comparisons between psychiatric disorders in the EPICure2 and EPICure cohorts were made using the subsample of EP children and their contemporaneous term-born peers (born at 22-25 completed weeks of gestation in England). Differences between EP and term-born children in each cohort and between EP children in EPICure2 and EPICure were again assessed using binary logistic regression analysis. Clinically important covariates were adjusted for in the between-cohort models, including sex, gestational age, birthweight *z* score, IMD at 11 years, multiple births, maternal age at birth, age at assessment, and severe disability. Children with missing data for confounders were excluded from the analysis (EPICure EP vs term: EP, n = 2; term, n = 29; EPICure2 EP vs term: EP, n = 1; term, n = 3; EPICure2 vs EPICure: EPICure2 EP, n = 3; EPICure EP, n = 5). In addition, maternal ethnicity was included as a covariate for analysis of difference in any psychiatric disorder between EP children in the 2 cohorts. It was not possible to include ethnicity in analyses of between-group differences in any emotional disorder, any ADHD, any ASD, or any conduct disorder because of the smaller numbers of children with these diagnoses and concerns about model instability.

## Results

### EPICure2 Cohort

Of the 200 EP and 143 term-born children assessed at 11 years of age in the EPICure2 study, 72.5% (145/200) and 68.5% (98/143) had complete DAWBA data for research diagnoses to be assigned (parent DAWBA with or without teacher DAWBA). There were no significant differences in response rate between EP and term-born children. The number of children assigned a research diagnosis for each of the summary categories varied slightly because of incomplete data. As symptom pervasiveness across multiple settings (eg, home and school) is required for ADHD, diagnoses were assigned only where children had both parent and teacher DAWBA complete (EP, 52.5% [105/200]; term, 53.8% [77/143]).

### Drop-Out Analysis

Children in the EPICure2 cohort with sufficient data for assignment of DAWBA diagnoses (“DAWBA”) were compared with those with no DAWBA or insufficient data for diagnosis (“no DAWBA”). Children with DAWBA were 0.2 years younger than those with no DAWBA (11.7 vs 11.9 years, t[199] = 2.584, *p* = .01) and had a higher mean IMD decile (ie, were less deprived) at both 11 years (IMD = 5.7 vs 4.3, *t*[331] = –4.276, *p* <.001) and at birth (IMD = 4.7 vs 3.9, *t*[196] = –2.071, *p =* .04). There was a statistically significant difference in the maternal ethnicity of children with DAWBA compared with no DAWBA: 81.4% of children born to White mothers had complete DAWBA compared with 50.0%, 62.2% and 72.7% of children born to Asian, Black, and Other/Mixed ethnicity mothers (DAWBA vs no DAWBA maternal ethnicity, *p =* .002). There were no significant differences between DAWBA and no DAWBA groups in sex, severe disability, gestational age, birthweight, proportion of multiple births, or maternal age at birth (Table S1, available online).

### Demographic Characteristics

Of the children who were assigned diagnoses, similar proportions of EP and term-born children in the EPICure2 cohort were male (49.0% EP and 45.9% term-born), had a similar mean age at assessment (EP children, 11.8 years [SD 0.6]; term-born, 11.7 years [0.6]), and both groups had a mean IMD decile at 11 years of 5.7 (SD = 2.8) (Table 1). As expected, there were significant differences in the proportion of EP and term-born children with severe disability (EP, 15.2%; term-born, 0%). Perinatal characteristics for the EP children are shown in Table 1.

**Table 1:** Demographic and Perinatal Characteristics of Extremely Preterm and Term-Born Children Within the EPICure2 and EPICure Cohorts

	EPICure2 (22-26 wk)			EPICure2 (England, 22-25 wk)			EPICure (England, 22-25 wk)			EPICure2 vs EPICure, 22-25 wk
	EP (A)	Term-born (B)	A vs B, *p*^a^	EP (C)	Term-born (D)	C vs D, *p*^a^	EP (E)	Term-born (F)	E vs F, *p*^a^	C vs E, *p*^a^
	n = 145	n = 98		n = 76	n = 98		n = 161	n = 143		
Sex, male, % (n)	49.0 (71)	45.9 (45)	.64	50.0 (38)	45.9 (45)	.59	44.7 (72)	42.7 (61)	.72	.45
Age at assessment, y, mean (SD)	11.8 (0.6)	11.7 (0.6)	.64	11.8 (0.5)	11.7 (0.6)	.48	10.8 (0.4)	10.9 (0.5)	.22	**<.001**
IMD at 11 y, mean (*r*)	5.7 (1-10), n = 140	5.7 (1-10), n = 95	.84	5.4 (1-10), n = 75	5.7 (1-10), n = 95	.46	5.2 (1-10), n = 159	5.8 (1-10), n = 114	.11	.62
Severe disability at 11 y, % (n)^b^	15.2 (22)	0.0 (0)	**<.001**	22.4 (17)	0.0 (0)	**<.001**	17.4 (28)	0.0 (0)	**<.001**	.36
										
Gestational age, mean (SD)	25.6 (1.0)	—		24.9 (0.8)	—		24.9 (0.7)	—		.78
≤23 wk, % (n)	8.3 (12)	—		15.8 (12)	—		10.6 (17)	—		.45
24 wk, % (n)	14.5 (21)	—		27.6 (21)	—		32.9 (53)	—		
25, % (n)	29.7 (43)	—		56.6 (43)	—		56.5 (91)	—		
26, wk, % (n)	47.6 (69)	—		—	—		—	—		
Birthweight (g), mean (*r*)	822 (479-1195)	—		745 (479-1059)	—		750 (480-1040)	—		.78
Birthweight *z* score, mean (SD)	−0.2 (0.8)	—		−0.1 (0.7)	—		−0.1 (0.8)	—		.77
Multiple births, % (n)	25.5 (37)	—		23.7 (18)	—		28.0 (45)	—		.49
Maternal age, mean (*r*)	31 (13-54)	—		31 (16-54)	—		29 (14-43)	—		**.03**
IMD at birth, mean (*r*)	4.8 (1-10) (n = 144)	—		4.7 (1-10)	—		—	—		—
Maternal ethnicity: White, % (n)	67.1 (96)	—		67.6 (50)	—		79.4 (127)	—		**.04**
Asian, % (n)	11.2 (16)	—		10.8 (8)	—		5.6 (9)	—		
Black, % (n)	16.1 (23)	—		16.2 (12)	—		14.4 (23)	—		
Other/mixed, % (n)	5.6 (8)	—		5.4 (4)	—		0.6 (1)	—		

Note: Boldface type indicates significant *p* value (<.05). EP = extremely preterm; IMD = index of multiple deprivation.

a *p* Value from χ^2^ test/Fisher’s exact test/Fisher–Freeman–Halton exact test for categorical outcomes and independent-samples *t* test for continuous outcomes.

b Severe disability (one or more of the following: Mental Processing Index >3 SD below term-born mean (<67), Gross Motor Function Classification System (GMFCS)/Manual Ability Classification System (MACS) ≥3, no useful hearing with aids, no useful vision or sees only gross light/movement).

### EPICure2 Psychiatric Disorders

**Figure 1:**
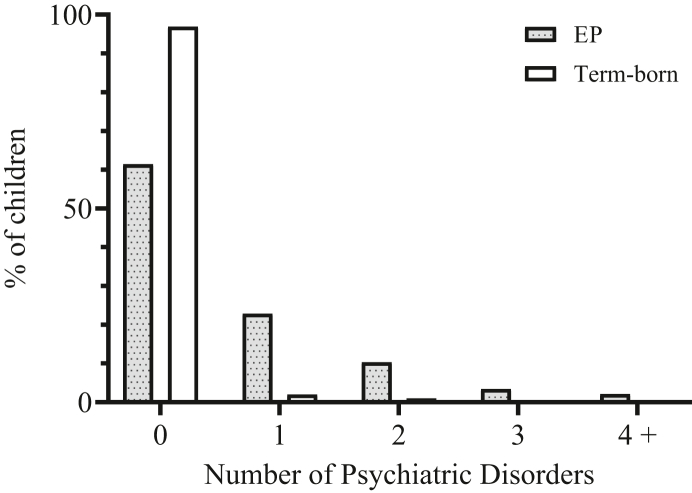
Number of *DSM-IV* Psychiatric Disorders for Extremely Preterm (EP, 22-26 Weeks of Gestational Age) and Term-Born Children in the EPICure2 Cohort at 11 Years

EP children in the EPICure2 cohort had 20.5 times the odds (95% CI = 6.2-67.9) of being assigned a diagnosis of any psychiatric disorder compared with their term-born peers (Table 2). This reduced to 15.1 (95% CI = 4.4-51.1) after adjusting for sex, IMD, and severe disability at 11 years. As well as having higher odds of any disorder, more EP children were assigned multiple disorders: 10.3% of EP children had diagnoses of 2 disorders, a further 3.4% had 3 diagnoses, and 2.1% had 4 or more diagnoses. Only 1 term-born child had more than 1 diagnosis (Figure 1).

**Table 2:** Prevalence of *DSM-IV* Psychiatric Disorders in Extremely Preterm (22-26 Weeks of Gestational Age) and Term-Born Children in the EPICure2 Cohort at Age 11 Years

EPICure2 Cohort (22-26 wk)	EP % (n/N)	Term-born % (n/N)	Unadjusted OR (95% CI)	*p*	Adjusted^a^ OR (95% CI)	*p*
Any psychiatric disorder	39.3 (57/145)	3.1 (3/98)	20.5 (6.2-67.9)	**<.001**	15.1 (4.4-51.1)	**<.001**
Any emotional disorder	14.6 (21/144)	2.0 (2/98)	8.2 (1.9-35.8)	**.005**	7.3 (1.6-32.7)	**.009**
Any conduct disorder	6.3 (9/143)	0.0 (0/98)	—	**.01**^c^		
Any ADHD^b^	21.9 (23/105)	2.6 (2/77)	10.5 (2.4-46.1)	**.002**	7.2 (1.5-33.6)	**.01**
Any ASD	18.9 (27/143)	0.0 (0/97)	—	**<.001**^d^		

Note: Boldface type indicates significant *p* value (<.05). ADHD = attention-deficit/hyperactivity disorder; ASD = autism spectrum disorder; EP = extremely preterm; OR = odds ratio

a Binary logistic regression, adjusted for sex, severe disability (one or more of the following: Mental Processing Index >3 SD below term-born mean (<67), Gross Motor Function Classification System (GMFCS)/Manual Ability Classification System (MACS) ≥3, no useful hearing with aids, no useful vision or sees only gross light/movement) and IMD at 11 years.

b Clinical ratings included only where both parent and teacher Developmental and Wellbeing Assessment (DAWBA) were complete to evaluate pervasiveness across settings.

c Fisher’s exact test.

d χ^2^ Test.

EP children were significantly more likely than term-born children to be assigned a diagnosis of emotional disorder (14.6% vs 2.0%; OR = 8.2, 95% CI = 1.9-35.8), conduct disorder (6.3% vs 0.0%; Fisher’s exact, *p =* .01), ADHD (21.9% vs 2.6%, OR = 10.5, 95% CI = 2.4-46.1), and ASD (18.9% vs 0.0%; Fisher’s exact, *p <* .001). The OR for both emotional disorders and ADHD decreased after adjustment for confounders (Table 2). No term-born children had diagnoses of conduct disorder or ASD.

Sensitivity analysis excluding children with severe cognitive impairment (IQ <70) reduced the number and percentage of EP children with any psychiatric disorder from 57 in 145 (39.3%) to 39 in 122 (32.0%), but did not significantly alter the results (Table S2, available online).

### Change Over Time: EPICure2 vs EPICure

There were 112 EP children in the EPICure2 cohort and 176 EP children in the EPICure cohort who were born between 22 and 25 completed weeks of gestation to mothers residing in England at the time of birth. Including the 143 and 153 term-born children in the EPICure2 and EPICure cohorts, respectively, these 584 children made up the subsample used for analyses of differences between the 2 cohorts.

Of this subsample, rates of complete DAWBA data were lower for EP and term-born children in EPICure2 than EPICure: 67.9% (76 of 112) of EPICure2 EP and 68.5% (98 of 143) of EPICure2 term-born children had complete DAWBA data, compared with 91.5% (161 of 176) of EPICure EP and 93.5% (143 of 153) of EPICure term-born children. The proportion of children with complete parent and teacher DAWBAs required for assignment of ADHD diagnoses was lower: EPICure2 EP 50.0% (56 of 112) and term-born 53.8% (77 of 143), and for EPICure EP 83.5% (147 of 176) and term 90.2% (138 of 153). No differences were seen in response rate between the subsample of EP and term-born children within each cohort.

### Demographic Characteristics

There were no differences in any demographic characteristics between the subsamples of EP and term-born children in the EPICure2 or EPICure cohort, except for severe disability at 11 years (Table 1).

Similar proportions of EP children in EPICure2 and EPICure were male. The majority of EP children in both cohorts were born at 25 weeks (EPICure2, 56.6%; EPICure, 56.5%). A total of 27.6% and 15.8% of EP children in EPICure2 and 32.9% and 10.6% in EPICure were born at 24 and 23 weeks of gestation, respectively. Birthweight and birthweight *z* scores, mean IMD decile at 11 years, percentage of children with severe disability, and proportion of children born as part of multiple births was similar for EP children in both cohorts (Table 1). EP children in EPICure2 were assessed at a significantly older mean age than those in EPICure (11.8 years vs 10.8 years, *t*[117] = 15.296, *p <* .001) and were also born to mothers with a higher mean age (29 years vs 31 years, *t*[234] = 2.225, *p =* .03). There were differences in maternal ethnicity between EP children in EPICure2 and EPICure (Fisher–Freeman–Halton Exact Test (*p =* .04). There were more mothers of Asian, Black, and Other/Mixed ethnicity in the EPICure2 cohort compared with the EPICure cohort, which is reflective of changes in the UK population over a similar time period.^37^

### Drop-Out Analysis

Among the subsamples, children with complete DAWBA (“DAWBA”) in EPICure2 were assessed at a slightly lower mean age of 11.8 years compared with those with incomplete/no DAWBA (“no DAWBA”), who were assessed at a mean age of 11.9 years (mean difference 0.1 years, *t*[253] = 2.105, *p =* .04). Similarly, the DAWBA group in EPICure were assessed at a lower mean than the no DAWBA group (10.9 vs 11.2 years, *t*[327] = 3.027, *p =* .003). Differences were also seen in socioeconomic status at 11 years between DAWBA and no DAWBA children in EPICure2: the mean IMD at 11 years of those with DAWBA was higher compared with no DAWBA (5.6 vs 4.3, *t*[247] = –3.508, *p <* .001). This difference was also present at birth. EPICure children with DAWBA also had a higher mean IMD at 11 years than no DAWBA children (5.4 vs 4.3), but this difference was not statistically significant. No IMD data were available for EPICure children at birth. Differences were also observed in the distribution of maternal ethnicity between EP children with complete DAWBA and those with no DAWBA in the EPICure2 cohort. Of the children born to White mothers, 82.0% had complete DAWBA, compared with 38.1% of children born to Asian mothers, 52.2% to Black mothers and 80.0% to Other/Mixed ethnicity mothers. The distribution of maternal ethnicity was similar for children across ethnic group for the EPICure cohort, which may be associated with the higher overall response rate for this cohort. No significant differences were observed in sex, severe disability, gestational age, birthweight, percentage of multiple births, and maternal age at birth between EP and term-born children with DAWBA compared with no DAWBA in the either cohort (Table S1, available online).

### EPICure2 vs EPICure Psychiatric Disorders

In accordance with findings from the EPICure2 cohort, EP children in the EPICure and EPICure2 subsamples also had a significantly higher prevalence of any psychiatric disorder, emotional disorders, ADHD, and ASD than their contemporaneous term-born peers (Table 3). In contrast, there were no differences in the rate of conduct disorder between EP and term-born children in EPICure, whereas 5.3% of EPICure2 EP children (22-25 weeks of gestation) had conduct disorder diagnoses compared with 0% of term-born peers (*p =* .03).

**Table 3:** Prevalence of *DSM-IV* Psychiatric Disorders in Extremely Preterm (22-25 Weeks of Gestational Age) and Term-Born Children in the EPICure2 Cohort at Age 11 Years Compared With EPICure (22-25 Weeks) and Term-Born Children

	EPICure (England, 22-25 wk)		EPICure2 (England, 22-25 wk)		EPICure EP vs term-born (A vs B)				EPICure2 EP vs term-born (C vs D)				EPICure2 vs EPICure (C vs A)			
DAWBA Assigned Research Diagnosis	EP (A), N = 161 % (n)	Term-born (B), N = 143 % (n)	EP (C), N = 76 % (n)	Term-born (D), N = 98 % (n)	Unadj OR (95% CI)	*p*	Adj OR (95% CI)^a^	*p*	Unadj OR (95% CI)	*p*	Adj OR (95% CI)^a^	*p*	Unadj OR (95% CI)	*p*	Adj OR (95% CI)^b^	*p*
Any psychiatric disorder	26.1 (42)	9.1 (13)	38.2 (29)	3.1 (3)	3.5 (1.8-6.9)	**<.001**	2.7 (1.2-5.9)	**.01**	19.5 (5.7-67.5)	**<.001**	13.2 (3.6-48.7)	**<.001**	1.7 (1.0-3.1)	.06	1.2 (0.5-2.6)^c^	.69
																
Any emotional disorder	9.3 (15)	2.1 (3)	13.2 (10)	2.0 (2)	4.8 (1.4-16.9)	**.02**	5.2 (1.1-24.2)	**.03**	7.3 (1.5-34.3)	**.01**	7.4 (1.5-36.3)	**.01**	1.5 (0.6-3.5)	.37	1.1 (0.4-3.2)	.85
			**(N = 75)**													
Any conduct disorder	5.6 (9)	6.3 (9)	5.3 (4)	0.0 (0)	0.9 (0.3-2.3)	.80	0.5 (0.1-1.6)	.23	—	**.03**^d^	—	—	1.0 (0.3-3.2)	.94	1.3 (0.2-6.7)	.78
	**(N = 147)**	**(N = 138)**	**(N = 56)**	**(N = 77)**												
Any ADHD (excluding other hyperactivity)	12.2 (18)	2.9 (4)	23.2 (13)	2.6 (2)	4.7 (1.5-14.2)	**.006**	4.4 (1.2-16.6)	**.03**	11.3 (2.4-52.6)	**.002**	9.0 (1.7-48.5)	**.01**	2.2 (1.0-4.8)	.06	1.5 (0.5-4.2)	.49
			(N = 75)													
Any ASD	8.7 (14)	0.0 (0)	21.3 (16)	0.0 (0)	—	**<.001**^d^	—	—	—	**<.001**^d^	—	—	2.8 (1.3-6.2)	**.008**	1.6 (0.6-4.6)	.36

Note: Boldface type indicates significant *p* value (<.05). ADHD = attention-deficit/hyperactivity disorder; Adj = ASD = autism spectrum disorder; EP = extremely preterm; OR = odds ratio; Unadj = unadjusted.

a Binary logistic regression, adjusted for sex, severe disability (one or more of the following: Mental Processing Index >3 SD below term-born mean (<67), Gross Motor Function Classification System (GMFCS)/Manual Ability Classification System (MACS) ≥3, no useful hearing with aids, no useful vision or sees only gross light/movement) and IMD at 11 years.

b Binary logistic regression, adjusted for sex, gestational age, birthweight z score, Index of Multiple Deprivation (IMD) at 11 years, multiple births, maternal age at birth, age at assessment (<11 and ≥11 years), and severe disability.

c Any psychiatric disorder binary logistic regression also adjusted for ethnicity (White vs Asian/Black/Mixed/Other ethnicity)

d Fisher’s exact/ χ^2^test as logistic regression not possible due to 0 term-born children with ASD.

In all, 38.2% of EPICure2 EP children and 26.1% of EPICure EP children had a DAWBA psychiatric diagnosis. Comparing EP children across the 2 cohorts, EP children in EPICure2 had higher unadjusted odds of any psychiatric diagnosis than EP children in EPICure (OR = 1.7, 95% CI = 1.0-3.1); however, this was not statistically significant. EP children in the EPICure2 cohort had an almost 3 times higher odds of ASD diagnosis than EP children in EPICure (unadjusted OR = 2.8, 95% CI = 1.3-6.2, *p =* .008); however, this reduced to 1.6 (95% CI = 0.6-4.6) and was non-significant after adjustment for confounders. No significant differences were observed in the rates of diagnosis of emotional disorders, conduct disorders, and ADHD between EP children in EPICure2 and EPICure (Table 3).

Sensitivity analysis excluding children with severe cognitive impairment (IQ <70) reduced the differences between the cohorts in ASD diagnosis (EPICure2 vs EPICure unadjusted OR = 1.6, 95% CI = 0.6-4.8) and was non-significant. The odds ratios for emotional disorders, conduct disorders and ADHD all remained non-significant (Table S3, available online).

## Discussion

This is the first study to investigate change in psychiatric disorders over time between 2 prospective population-based cohorts of EP children born in different decades. The EPICure2 study of children born between 22 and 26 weeks of gestation in England in 2006 demonstrated that children born EP were significantly more likely to have diagnoses of any psychiatric disorder, emotional disorder, conduct disorder, ADHD, and ASD than their contemporaneous term-born peers. EP children also had a greater number of psychiatric disorders than term-born children.

Comparing EP children in EPICure2 with those born in the earlier EPICure cohort of births in 1995 using a subsample of children born at 22 to 25 weeks of gestation to mothers residing in England, there were no significant differences in the proportion of children with any psychiatric disorder, emotional disorder, conduct disorder, or ADHD. Relative to EPICure, significantly more EP children in EPICure2 had diagnoses of ASD; however, this difference was not significant after adjustment. Increased survival for infants born EP has not therefore translated into improvements in mental health outcomes.

Comparing findings to the existing literature, rates of any psychiatric disorder in the EPICure2 cohort EP children are higher than those reported in the VIBeS cohort at both 7 and 13 years of age (39.3% vs 24% and 27.3%), which also used the DAWBA.^16^^,^^17^ This could be because the EPICure2 cohort were more immature at birth (mean GA: EPICure2, 25.6; VIBeS, 27.5 weeks). However, rates of diagnoses were more similar between the EPICure EP children (22-25 weeks, mean GA 24.9 weeks) and the VIBeS cohort (EPICure 26.7% vs VIBeS 24%), making gestational age a less likely cause of differences. Rates of ADHD were higher for EP children in the EPICure2 cohort than those in the VIBeS and ELGAN cohorts (EPICure2, 22%; ELGAN, 18%, VIBeS at 13 years, 10%), but again rates in the EPICure cohort were comparable (12%).^17^^,^^19^ The reported prevalence of ASD was also higher for both EPICure cohorts than the VIBeS cohort, EPICure2 more so than EPICure. Rates of emotional disorders, however, appeared to increase with age at assessment. The VIBeS cohort at 7 years reported 11% of VP/VLBW children with anxiety and less than 1% with mood disorders,^16^ and this increased to 14% and 2%, respectively, at 13 years.^17^ A total of 14% of EP children in EPICure2 had assigned diagnoses of emotional disorders; and the highest rates were reported at 15 years by the ELGAN cohort, of whom 16.5% of EP children were reported to have anxiety disorders and another 5% to have mood disorders.^19^

In contrast, the differences were reversed when considering term-born children. Term-born children in the VIBeS cohort had higher rates of any psychiatric diagnosis compared to EPICure2 (VIBeS, 9% at 7 years^16^ and 6.1 % at 13 years^17^; EPICure2, 3.1%), further increasing the differences seen between EP and term-born children in the EPICure2 cohort. The rate of disorders in term-born children was similar between the VIBeS cohort at 7 years of age and the EPICure cohort (EPICure 9.1%). No term-born children were assessed as part of the ELGAN study. Furthermore, the rate of psychiatric disorders in the term-born children in both EPICure cohorts is lower than expected for the general population: 9.5% of individuals 5 to 10 years of age and 14.4% of those 11 to 16 years of age in England in 2017 were estimated to have a mental disorder following assessment with the DAWBA in a stratified probability sample,^38^ compared with 9.1% and 3.1% for EPICure and EPICure2 term-born children, respectively. This may suggest that term-born children in the EPICure studies, particularly in EPICure2, had better mental health on average, which may have inflated differences observed between EP and term-born children. This could be a result of term-born children being recruited primarily from mainstream schools. However, rates of psychiatric disorders in EP children are much greater than population figures across all types of disorders studied except conduct disorder (general population estimate, 4.6%).^38^ This suggests that significant differences would remain even relative to population levels.

Despite differences in the proportion of EP children with psychiatric diagnoses between EPICure2 and EPICure, none were statistically significant after adjusting for confounders to account for differences in key clinical, ethnic, and socio-economic characteristics between the cohorts. The stability of psychiatric outcomes for EP children over time is in keeping with reports of no change in neurodevelopmental^25^ outcomes in the same children at 11 years, as well as in other consecutive cohort studies.^26^^,^^39^ It is also in keeping with meta-analysis results that showed no effect of birth year on mental health difficulties in ELBW survivors.^5^

The lack of improvement in mental health and other outcomes over time for children born EP could be explained in 2 ways. First, the increased survival of EP children may result in infants born at more immature gestational ages and with more neonatal complications surviving, when previously they would have died during the neonatal period. It is reasonable to expect that these more immature and sicker babies would have worse outcomes than those born at more mature gestational ages, even within the EP population. This shift may therefore be concealing improvements at more mature gestational ages as more immature infants survive. This is supported by the meta-regression finding by Kaempf *et al.*,^24^ who demonstrated improvements in neurodevelopmental outcomes when gestational age was controlled for, due to improvements at 25 and 26 weeks of gestational age. Indeed, there was a 5.2% increase in children born at 23 weeks in the EPICure2 cohort relative to the EPICure cohort, although the proportions born at 25 weeks were very similar (56.6% vs 56.5%). However, there was no significant difference in the proportion of infants with major neonatal morbidities between the EPICure2 and EPICure cohorts. Even in the absence of significant neonatal morbidities, brain growth and development could also be affected to a greater extent at more immature gestational ages, potentially increasing the risk of later psychiatric disorders. Week of gestational age was included as a confounder in our regression models to adjust for any differences between the cohorts; however, this may not fully adjust for differences related to neonatal complications as a result of gestational age. It was not possible to conduct subgroup analyses by week of gestational age because of our sample size.

Secondly, there is a general population trend toward increased childhood mental health problems^40^ and, in particular, ASD.^41^ This population increase may be mirrored or exacerbated in the EP population, counterbalancing any improvements related to better care. However, the opposite was observed in the rate of psychiatric disorders in the 2 term-born groups, with the EPICure2 term-born children having lower rates of any psychiatric disorder and emotional disorders/conduct disorders/ADHD/ASD than EPICure term-born children, making this explanation less likely. The increase in rates of ASD diagnosis in the EPICure2 cohort compared to that in EPICure is in keeping with increases in the general population,^41^ despite both cohorts being evaluated using the same *DSM-IV* diagnostic criteria and by experienced child and adolescent psychiatrists blinded to birth status. However, differences between the cohorts were non-significant after adjusting for confounders and on sensitivity analysis excluding children with severe cognitive impairment, further supporting the former theory that sicker and more immature infants may be surviving. No term-born children were assigned a diagnosis of ASD in either cohort, which is unexpected, given the sample size and population prevalence of at least 1%,^42^ but again this could reflect the recruitment of term-born children only from mainstream schools.

One major strength of this study is the prospective longitudinal design and follow-up of 2 population-based cohorts alongside contemporaneous term-born children, using the same diagnostic measure of psychiatric disorders. This consistency in study design allowed robust comparison of outcomes over time. In addition, recruiting term-born comparison children from the same school as EP children reduces bias in socioeconomic status and childhood educational experience. However, there were difficulties with retention, a frequent finding in longitudinal studies. The EPICure2 EP sample assessed comprised 19% of the whole cohort of long-term survivors^25^ and had a lower response rate than the EPICure sample. This is a limitation of the study; however, despite this, the only differences between the children assessed in the EPICure and EPICure2 were age at assessment, maternal ethnicity, and maternal age, which were included as covariates in the regression analysis. The difference in age at assessment is a further limitation, particularly because the 1-year difference between cohorts falls across the transition between primary and secondary school in the UK education system. This change in school setting may contribute to changes in mental health. Difficulties, particularly in social and communication skills, may be exacerbated by the transition to secondary school, which could be an additional contributing factor to the higher rates of ASD seen in EPICure2 and explained by the reduced differences after adjusting for age at assessment. The difference in maternal ethnicity between EP children in the EPICure2 and EPICure cohorts is reflective of changes in ethnicity across England and Wales from census data collected in 1991, 2001, and 2011, which showed an increase in ethnic diversity.^37^ EP children in EPICure2 born to mothers of Asian or Black ethnicity were less likely to have complete DAWBA compared to children born to White mothers, which is a limitation of the study, and the subsequent effect on rates of psychiatric disorders is therefore unknown. The DAWBA was available to complete only in English, and so this may potentially reflect a language barrier to completion for the EPICure2 cohort that has occurred with the increase in ethnic diversity within England over time. No significant differences were seen in maternal ethnicity between those with complete DAWBA and no DAWBA in the EPICure cohort; however, a greater proportion of this cohort were born to White mothers compared to other ethnicities, and there was a higher overall response rate for this cohort. Maternal ethnicity was added as a covariate to the regression model for differences in any psychiatric disorder between the 2 cohorts and did not significantly change the odds ratios or confidence intervals. On this basis, it is thought that maternal ethnicity has a minimal effect on the risk of any psychiatric disorder following EP birth in the 2 UK samples. In addition, children with complete DAWBA in both cohorts were of higher socioeconomic status than those who were lost to follow-up, which may affect the external validity of the study. However, the selective loss to follow-up of children from lower socioeconomic status was the same in both cohorts, limiting the impact of this on the results of analysis of change in outcomes over time.

Finally, poor maternal mental health during pregnancy^43^ and childhood^44^ has been shown to be associated with increased risk of childhood behavioral and developmental disorders. Poor antenatal mental health is also associated with an increased risk of preterm delivery.^45^ Furthermore, having a VP infant also predisposes mothers to higher levels of psychological distress, which is sustained throughout their child’s life into adolescence,^46^ further increasing the risk of maternal mental health problems. Maternal mental health data were not collected, which is a limitation of this study and an area for further research.

In summary, children born EP in both the EPICure2 and EPICure cohorts consistently had higher rates of psychiatric disorders than their term-born peers, particularly emotional disorders, ADHD, and ASD. The impact of EP birth on future psychiatric health is an important factor for health care professionals to consider, and must not be overlooked relative to neurodevelopmental and cognitive outcomes, which are often more closely followed-up in early childhood. There has been no improvement in psychiatric outcomes between the 2 cohorts born 11 years apart. Further research is needed to ascertain whether the lack of improvement in outcomes is due to sicker infants surviving to discharge, the impact of altered *ex utero* brain growth and development, part of worsening mental health in the general population, or a lag in improvement in long-term outcomes that has not yet been observed following improved neonatal care. In addition, research into strategies to support children born EP with psychiatric disorders is pertinent.

## CRediT authorship contribution statement

**Jennifer Larsen:** Writing – review & editing, Writing – original draft, Project administration, Methodology, Formal analysis, Data curation. **Josephine Holland:** Writing – review & editing, Methodology, Formal analysis. **Puja Kochhar:** Writing – review & editing, Methodology, Formal analysis. **Dieter Wolke:** Writing – review & editing, Conceptualization, Formal analysis, Funding acquisition, Methodology. **Elizabeth S. Draper:** Writing – review & editing, Supervision, Formal analysis. **Neil Marlow:** Writing – review & editing, Supervision, Methodology, Funding acquisition, Conceptualization, Formal analysis. **Samantha Johnson:** Writing – review & editing, Supervision, Methodology, Conceptualization, Formal analysis, Funding acquisition.
