# Supplementary material for: Comparing the Prevalence of Psychiatric Disorders in Cohorts of Children Born Extremely Preterm in 1995 and 2006: The EPICure Studies
Source: JAACAP Open. 2024 Mar 26;2(3):217–28. doi: 10.1016/j.jaacop.2024.02.005 (PMC11372438; doi:10.1016/j.jaacop.2024.02.005)
Supplement: Supplemental Material [file mmc1.pdf]

Figure S1: DSM-IV Psychiatric disorders assigned by the Developmental and Well-Being Assessment (DAWBA) and included in 'any psychiatric disorder' and summary outcomes: emotional disorders, conduct disorders, attention-deficit/hyperactivity disorders and autism spectrum disorders.

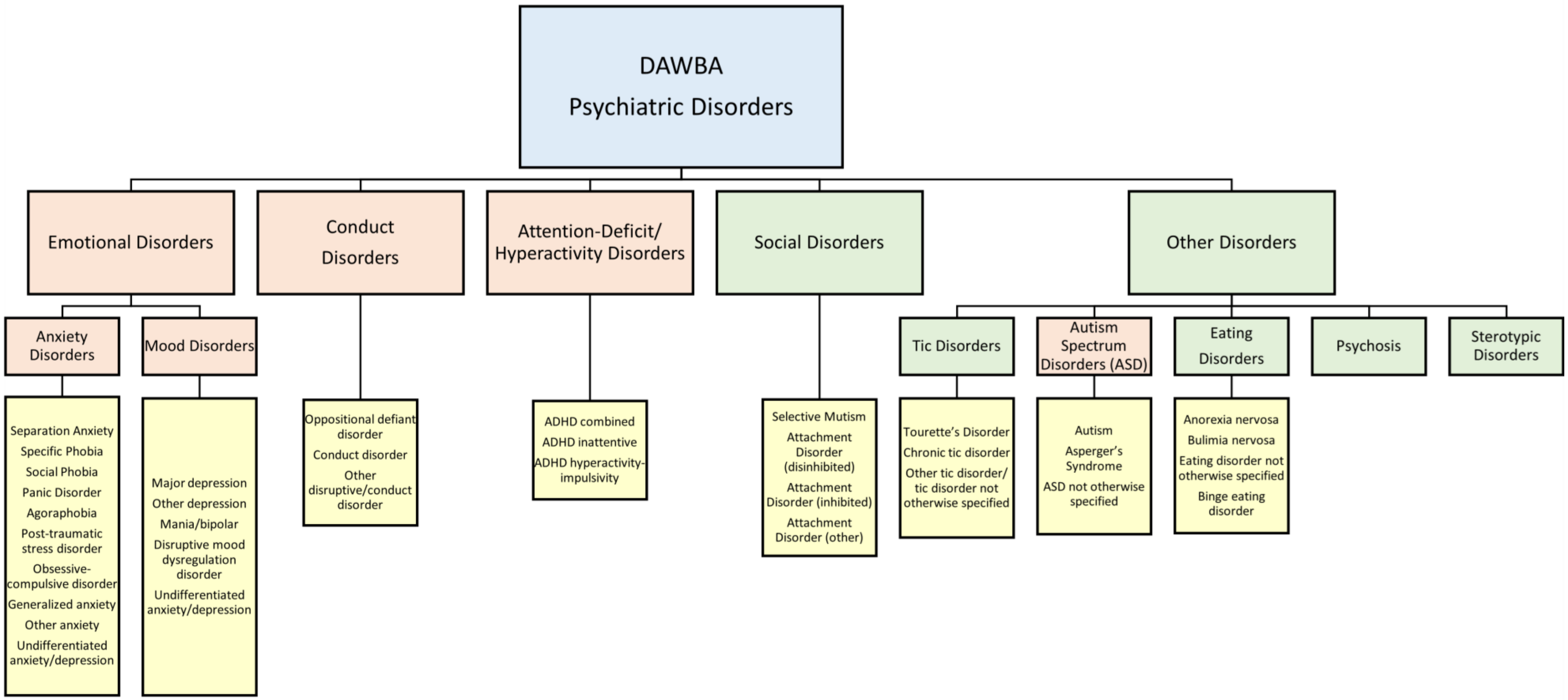

Table S1: Drop-out analysis of children with complete DAWBA data versus no DAWBA for EPICure2 (22-26 weeks [a] and term-born children[b]); EPICure2 (22-25 weeks [c] and term-born children [d]); and EPICure (22-25 weeks [e] and term-born children [f]).

	**EPICure2 (22-26 weeks and term-born)**			**EPICure2 (22-25 weeks, England and term-born)**			**EPICure (22-25 weeks, England and term-born)**		
	**DAWBA [A]**	**No DAWBA [B]**	**A vs B *P* value**	**DAWBA [C]**	**No DAWBA [D]**	**C vs D *P* value**	**DAWBA [E]**	**No DAWBA [F]**	**E vs F *P* value**
	N=243	N=100		N=174	N=81		N=304	N=25	
**Age at Assessment, mean (range)**	11.7 (10.4-13.2)	11.9 (10.5-13.0)	**0.01**	11.8 (10.4-13.2)	11.9 (10.5-13.0)	**0.04**	10.9 (9.8-12.3)	11.2 (10.2-12.0)	**0.003**
**Male Sex, % (n)**	47.7% (116)	47.0% (47)	0.90	47.7% (83)	44.4% (36)	0.63	43.8% (133)	44.0% (11)	0.98
**IMD at 11 years, mean (range)**	5.7 (1-10) [N=235]	4.3 (1-10) [N=98]	**<0.001**	5.6 (1-10) [N=170]	4.3 (1-10) [N=79]	**<0.001**	5.4 (1-10) [N=273]	4.3 (1-10) [N=24]	0.07
**Severe disability [a] at 11 years, % (n)**	9.1% (22)	14.0% (14)	0.17	9.8% (17)	14.8% (12)	0.24	9.2% (28)	16.0% (4)	0.29
	N=145	N=55		N=76	N=36		N=161	N=15	
**Gestational age, mean (SD)**	25.6 (1.0)	25.5 (0.9)	0.41	24.9 (0.8)	25.0 (0.7)	0.33	24.9 (0.7)	24.9 (0.8)	0.97
**≤23w %(n**	8.3% (12)	5.5% (3)	0.13	15.8% (12)	8.3% (3)	0.27	10.6% (17)	13.3% (2)	0.92
**24w %(n)**	14.5% (21)	12.7% (7)		27.6% (21)	19.4% (7)		32.9% (53)	33.3% (5)	
**25w %(n)**	29.7% (43)	47.3% (26)		56.6% (43)	72.2% (26)		56.5% (91)	53.3% (8)	
**26w %(n)**	47.6%% (69)	34.5% (19)		---	---		---	---	
**Birthweight (g), mean(range)**	822 (479-1195)	778 (540-1100)	0.06	745 (479-1059)	728 (546-990)	0.49	750 (480-1040)	706 (570-880)	0.14
**Birthweight z scores, mean (SD)**	-0.2 (0.8)	-0.4 (0.7)	0.07	-0.1 (0.7)	-0.4 (0.7)	0.10	-0.1 (0.8)	-0.4 (0.9)	0.12
**Multiple birth, %(n)**	25.5% (37)	21.8% (12)	0.59	23.7% (18)	25.0% (9)	0.88	28.0% (45)	40.0% (6)	0.38
**Maternal age, mean(range)**	31 (13-54)	31 (21-47)	0.87	31 (16-54)	31 (21-41)	0.79	29 (14-43)	27 (17-36)	0.17
**IMD at birth, mean (range)**	4.7 (1-10) [N=144]	3.9 (1-9) [N=54]	**0.04**	4.7 (1-10)	3.4 (1-9) [N=35]	**0.01**	---	---	
**Maternal Ethnicity: White**	67.1% (96)	40.0% (22)	**0.002**	67.6% (50)	30.6% (11)	**<0.001**	79.4% (127)	73.3% (11)	0.615
**Asian**	11.2% (16)	29.1% (16)		10.8% (8)	36.1% (13)		5.6% (9)	6.7% (1)	
**Black**	16.1% (23)	25.5% (14)		16.2% (12)	30.6% (11)		14.4% (23)	20.0% (3)	
**Other/Mixed**	5.6% (8)	5.5% (3)		5.4% (4)	2.8% (1)		0.6% (1)	0.0% (0)	

[a] severe disability (one or more of the following: Mental Processing Index >3 SD below control mean (<67), GMFCS/MACS ≥3, no useful hearing with aids, no useful vision or only sees gross light/movement)

Table S2: Prevalence of DSM-IV Psychiatric Disorders in extremely preterm (EP, 22-26 weeks of gestational age) and term-born children in the EPICure2 cohort at age 11 years, excluding children with severe cognitive impairment (IQ<70).

**DAWBA Assigned Research Diagnosis**	**EP (22-26 weeks)**	**Term-born**	**Unadjusted OR (95% CI)**	***P***	**Adjusted [a] OR (95% CI)**	***P***
**Any psychiatric disorder**	32.0% (39/122)	3.1% (95/98)	14.9 (4.4, 49.9)	**<0.001**	14.7 (4.3, 49.8)	**<0.001**
**Any emotional disorder**	12.4% (15/121)	2.0% (2/98)	6.8 (1.5, 30.5)	**0.01**	6.4 (1.4, 28.8)	**0.02**
**Any conduct disorder**	6.7% (8/120)	0.0% (0/98)	---	**0.009[b]**		
**Any ADHD**	16.5% (14/85)	2.6% (2/77)	7.4 (1.6, 33.7)	**0.01**	7.3 (1.6, 34.3)	**0.01**
**Any ASD**	10.8% (13/120)	0.0% (0/97)	---	**0.001[c]**		

[a] Binary logistic regression – adjusted for sex and IMD at 11 years [Not possible to add severe neurosensory impairment as covariate as model became unstable due to complete/quasi-complete separation]

[b] Fisher's Exact Test

[c] Chi^2-test

Table S3: Prevalence of DSM-IV Psychiatric Disorders in extremely preterm (EP, 22-25 weeks of gestational age) and term-born children in the EPICure2 cohort at age 11 years compared with EPICure (22-25 weeks') and term-born children, excluding children with cognitive impairment (IQ<70).

	**EPICure 22-25 weeks**		**EPICure2 22-25 weeks[$]**		**EPICure Index vs Controls [a vs. b]**				**EPICure2 Index vs Controls [c vs. d]**				**EPICure2 vs EPICure [c vs a]**			
**DAWBA Assigned Research Diagnosis**	EP [A] N=137	Term-born [B] N=143	EP [C] N=59	Term-born [D] N=98	Unadj OR (95%CI)	*P*	Adj OR (95% CI)[a]	*P*	Unadj OR (95%CI)	*P*	Adj OR (95% CI)[a]	*P*	Unadj OR (95%CI)	*P*	Adj OR (95% CI)[b]	*P*
**Excluding children with cognitive impairment (IQ<70)**																
**Any psychiatric disorder**	21.9% (30)	9.1% (13)	28.8% (17)	3.1% (3)	2.8 (1.4, 5.6)	**0.004**	2.9 (1.3, 6.3)	**0.008**	12.8 (3.6, 46.1)	**<0.001**	13.3 (3.6, 48.7)	**<0.001**	1.4 (0.7, 2.9)	0.30	1.1 (0.5, 2.7)[c]	0.82
**Any emotional disorder**	8.8% (12)	2.1% (3)	11.9% (7)	2.0% (2)	4.5 (1.2, 16.2)	**0.02**	5.1 (1.1, 23.7)	**0.04**	6.5 (1.3, 32.2)	**0.02**	6.2 (1.2, 31.0)	**0.03**	1.4 (0.5, 3.8)	0.50	1.9 (0.5, 6.6)	0.34
**Any conduct disorder**	3.6% (5)	6.3% (9)	5.2% (3)	0.0% (0)	0.6 (0.2, 1.7)	0.32	0.6 (0.2, 1.9)	0.36	---	0.05[d]	---	---	1.4 (0.3, 6.2)	0.63	1.3 (0.2, 7.6)	0.77
	[N=124]	[N=138]	[N=41]	[N=77]												
**Any ADHD**	10.5% (13)	2.9% (4)	17.1% (7)	2.6% (2)	3.9 (1.2, 12.4)	**0.02**	4.3 (1.1, 16.8)	**0.03**	7.7 (1.5, 39.1)	**0.01**	9.6 (1.8, 52.0)	**0.009**	1.8 (0.6, 4.8)	0.27	0.9 (0.3, 3.6)	0.99
			[N=58]	[N=97]												
**Any ASD**	6.6% (9)	0.0% (0)	10.3% (6)	0.0% (0)	---	**0.001[d]**	---	---	---	**0.002[d]**	---	---	1.6 (0.6, 4.8)	0.37	0.9 (0.3, 3.5)	0.92

ADHD=attention-deficit/hyperactivity disorder; ASD=autism spectrum disorder; EP=extremely preterm; OR=odds ratio

[a] adjusted for sex and IMD at 11 years

[b] adjusted for sex, gestational age, birthweight Z score, IMD at 11 years, multiple births, maternal age at birth and age at assessment (<11 vs. ≥11 years).

[c] Any psychiatric disorder binary logistic regression also adjusted for ethnicity (white vs Asian/Black/Mixed/Other ethnicity)

[d] Fisher's exact test
